# Analysis of the cervical microbiome and potential biomarkers from postpartum HIV-positive women displaying cervical intraepithelial lesions

**DOI:** 10.1038/s41598-017-17351-9

**Published:** 2017-12-12

**Authors:** Gislaine Curty, Raquel L. Costa, Juliana D. Siqueira, Angela I. Meyrelles, Elizabeth S. Machado, Esmeralda A. Soares, Marcelo A. Soares

**Affiliations:** 1 Programa de Oncovirologia, Instituto Nacional de Câncer, Rio de Janeiro, 20.231-050 Brazil; 2 Instituto de Ginecologia, Universidade Federal do Rio de Janeiro, Rio de Janeiro, Brazil; 3 Instituto de Puericultura e Pediatria Martagão Gesteira, Universidade Federal do Rio de Janeiro, Rio de Janeiro, Brazil

## Abstract

The cervical microbiota composition and diversity of HIV-positive women in the postpartum period is unknown. Using a high-throughput bacterial 16S rRNA gene sequencing, we identified four community state types (CSTs). CST III (*Lactobacillusdominant*) and CST IV (IV-A, IV-B.1, IV-B.2; high-diversity) were found in 41% and 59% of samples, respectively. We did not find association of any CST to postpartum period (six or twelve months), HPV infection or cytology (normal or lesion). However, five bacterial genera were associated with cervical lesions (*Gardnerella, Aerococcus, Schlegelella, Moryella* and *Bifidobacterium*), with significant odds ratio (OR) of 40 (2.28–706) for the presence of *Moryella* and 3.5 (1.36–8.9) for *Schlegelella*. Longitudinal analysis of samples at postpartum that regressed (lesion to normal), progressed (normal to lesion) and maintained the cytology (lesion or normal) evidenced *Gardnerella* with a significantly higher abundance in regressing lesions. In the current study, we report the first data on the cervical microbiota of HIV-positive women in the postpartum period. Consistent with previous studies of HIV-negative cohorts, HIV-positive women present a stable cervical microbiota of high-diversity in the postpartum period. Our results highlight that specific microbiota species may serve as sensors for changes in the cervical microenvironment associated with cervical lesions.

## Introduction

Cervical cancer is the fourth most common cancer among women worldwide, and over 500,000 new cases are diagnosed each year, leading to more than 200,000 deaths^1^. The primary factor associated with the development of cervical cancer is the persistent infection by the human papillomavirus (HPV). Additional factors correlated with HPV persistence include immunodeficiency caused by HIV, smoking, use of oral contraceptives and, more recently reported, cervicovaginal dysbiosis^2^.

The classically-defined normal cervicovaginal microbiota is dominated by one or more *Lactobacillus sp*. (*Lactobacillus crispatus*, *L*. *gasseri*, *L*. *iners* or *L*. *jensenii*). However, in a state of dysbiosis, there is a marked reduction of *Lactobacillus* and a high diversity of bacteria, with increased abundance of anaerobic bacterial species^3–5^. Cervicovaginal dysbiosis is asymptomatic in some cases, turning the definition of normal/dysbiotic microbiota a point of current discussion. However, many women with cervicovaginal dysbiosis develop symptomatic bacterial vaginosis (BV), characterized by vaginal discomfort and homogeneous malodorous vaginal discharge^6,7^. Both symptomatic and asymptomatic dysbiosis have been associated with the risk to preterm birth, acquisition of sexually transmitted infections (STIs) and increased risk of pelvic inflammatory disease^7–9^. In general, previous studies on cervical microbiome have described five community state types (CSTs), I to V^3^. Whereas CSTs I, II, III and V are dominated by *Lactobacillus* species (*L*. *crispatus*, *L*. *gasseri*, *L*. *iners* or *L*. *jensenii*, respectively), CST IV is characterized by a high diversity and predominance of anaerobic bacteria as *Gardnerella vaginalis*, *Atopobium vaginae*, *Prevotella sp*. and other anaerobic bacterial species^3^.

It has been shown that reduction of *Lactobacillus sp*., combined with increased diversity of cervicovaginal microbiota, are risk factors for HPV acquisition, persistence, development of cervical intraepithelial neoplasia (CIN) and cervical cancer^2^. A study conducted with Korean women with and without CIN showed that those carrying *Atopobium vaginae*, *Garderella vaginalis* and *L*. *iners* in the absence of *L*. *crispatus* had an almost 6x higher risk for CIN. That study also showed the synergistic effect of this microbial pattern and oncogenic HPV infection on a very high risk (odds ratio of 34.1) of CIN^10^. In a longitudinal study with samples collected over a 16-week period, women with highly diverse or *L*. *iners*-dominated cervicovaginal microbiota were more likely to be HPV-positive, while a microbiota dominated by *L*. *gasseri* was associated with more rapid clearance of HPV infection^11^.

The high diversity of cervicovaginal microbiota is strongly correlated with local inflammation and it is also thought to increase the risk for HIV acquisition^12^. It is known that HIV-positive women more often present with high-risk HPV infection and development of cervical disease^13^. However, little is known about the influence of the cervicovaginal dysbiosis in HIV/HPV coinfected women. Comparison of HIV-infected and HIV-uninfected women showed that microbiota dominated by *L*. *crispatus* are associated with a lower prevalence of HIV/STIs^14^ and with decreased prevalence of HPV in HIV-infected woman^15^. In contrast increased microbiome diversity has been demonstrated in high frequency among HIV-positive woman^14^. The cervicovaginal dysbiosis may also generally affect maternal health, leading to postpartum complications^16,17^. A longitudinal study of woman followed-up during pregnancy and postpartum showed that the cervicovaginal microbiota composition is modified at six weeks postpartum, with increased bacterial diversity and decrease in *L*. *crispatus* moieties^18^.

Based on this information, we hypothesize that postpartum HIV-positive women have a highly diverse cervicovaginal microbiota, which could be associated with an increased risk for developing persistent HPV infection and consequently CIN. Therefore, our study aimed to evaluate the postpartum cervical microbiota profiles of HIV-positive women displaying diverse cervical intraepithelial neoplasia.

## Results

### Sociodemographic and clinical baseline characteristics

The median age of participants was 28 years-old. Approximately 60% of the women never smoked, the median age of first sexual intercourse was 15 years-old, 70% had 3 or more sexual partners and 48% used hormonal contraceptives before pregnancy (Table 1). The median CD4^+^ T-cell count and HIV viral load values of the studied subjects were of 579 cells/*μ*l and 344 copies/ml at 6 months after labor, and of 548 cells/*μ*l and 409 copies/ml at 12 months after labor, respectively, and those differences were not significant. The frequency of HPV-negative women was significantly higher at 12 months than at 6 months postpartum (8% versus 37%). However, the frequency of HPV-positive women with lesions (LSIL/HSIL) was higher at 12 months compared to six months postpartum (Table 2).

**Table 1:** Sociodemographic characteristics of the studied participants.

Age (median, range)	28 (17–44)
Smoking (%, N/Total)	
Current smoker	15 (12/80)
Former smoker	26 (21/80)
Never smoker	58 (46/80)
N/A^1^	1 (1/80)
Median age of first sexual intercourse (range)	15 (9–25)
Number of sexual partners (%, N/Total)	
1 to 3 sexual partners	30 (24/80)
>3 sexual partners	70 (56/80)
Contraceptive methods (%, N/Total)	
Hormonal	48 (38/80)
Condom	33 (27/80)
Other	2 (2/80)
None	16 (13/80)

^1^N/A, not available.

**Table 2:** Frequency of cervical cytology and HPV status at six and 12 months postpartum.

		06 months % (n = 26)	12 months % (n = 54)	% N/Total	p-value^1^
Cervical Cytology	Normal	65 (17/26)	46 (25/54)	52 (42/80)	0.109
	Lesion	35 (9/26)	54 (29/54)	48 (38/80)	
HPV status	Positive	92 (24/26)	63 (34/54)	72 (58/80)	0.006
	Negative	8 (2/26)	37 (20/54)	28 (22/80)	
Cytology-HPV positive	Normal	58 (15/26)	15 (8/54)	29 (23/80)	0.003
	Lesion	38 (9/26)	48 (26/54)	43 (35/80)	
Cytology-HPV negative	Normal	8 (2/26)	31 (17/54)	24 (19/80)	0.556
	Lesion	0 (0/26)	6 (3/54)	4 (3/80)	

^1^Pearson’s chi-square test.

### Microbiome community diversity

**Figure 1:**
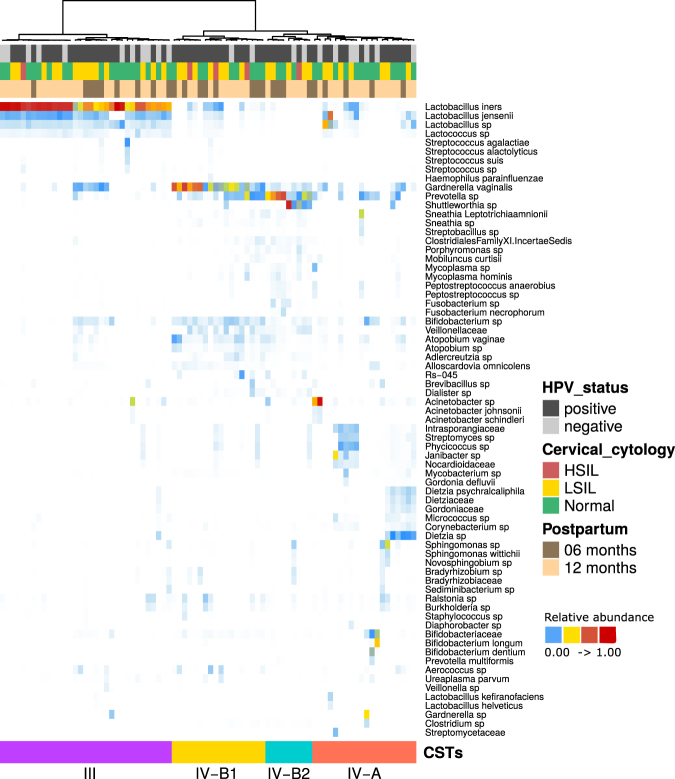
Heatmap generated by unsupervised hierarchical clustering analysis of cervical microbiomes of the studied participants. CSTs were defined using clustering based on Bray-Curtis dissimilarity and average linkage and are shown in color-coded groups at the bottom and also by the dendrogram at the top of the Figure. HPV status, cervical cytology and postpartum period are also color-coded according to the legend at the right of the Figure. CST III is *L*. *iners*-dominant; CST IV-A has a low proportion of *Lactobacillus* and a high diversity; CST IV-B.1 is *G*. *vaginalis*-dominant and CST IV-B.2 is *Prevotella*-dominant. All taxa shown in the graph presented relative abundance >1%.

After an initial unsupervised clustering analysis, four distinct CSTs were found and were named according to previous designations^11,19,20^ (Fig. 1 and Supplementary Figure 1A).

CST III is dominated by *L*. *iners*. CST IV was observed in a distinct split between IV-A and IV-B. CST IV-A showed a high bacterial diversity and a low abundance of *Lactobacillus*. However, we noticed a clear split within CST IV-B in two groups, one dominated by *Gardnerella* (which we named CST IV-B.1) and another dominated by *Prevotella* (CST IV-B.2) (Fig. 1 and Supplementary Figure 1B).

**Figure 2:**
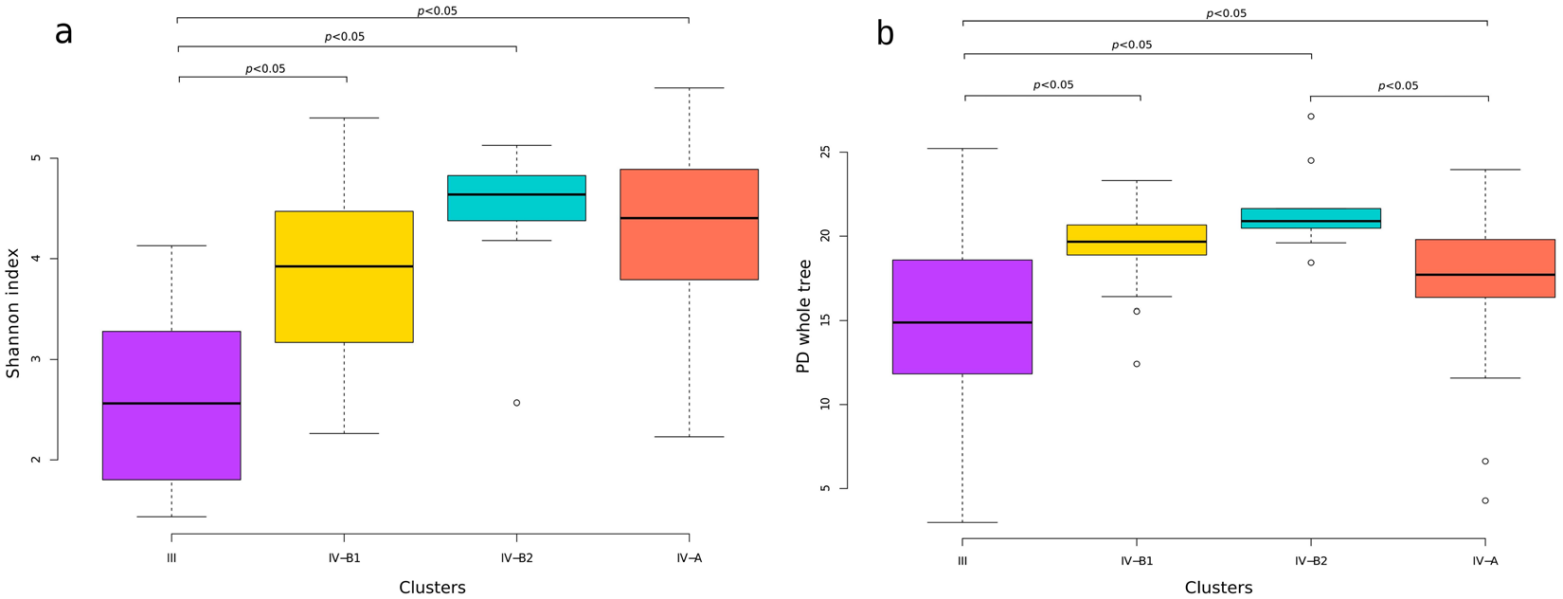
Box plot analysis of alpha diversity using the (**a**) Shannon index and (**b**) phylogenetic diversity. Analyses in (**a**) and (**b**) were performed for each CST defined. The Mann-Whitney test was carried out to compare diversities between each CST, and the significant p-values at the 0.05 confidence level are depicted at the horizontal lines above the graphs.

The Shannon’s diversity analysis showed a significantly lower diversity of CST III compared to the remaining CSTs (IV-A, IV-B.1, IV-B.2) (Fig. 2a). The Phylogenetic Diversity (PD) analysis corroborated those differences, and also suggested differences between CST IV-B2 and CST IV-A (Fig. 2b). These data show that our population under study predominantly carries a microbiota of high diversity and lacks *Lactobacillus*-dominated CSTs (except for *L*. *iners*), generally seen in other studies. Additionally, we performed bacterial profile analysis by reconstruction of the 16S gene to confirm the results obtained in the read analysis with QIIME, and the two methods showed a high degree of similarity, ranging from 61 to 86% (Supplementary Figure 2).

We next analyzed the distribution of the samples classified as CSTs III and IV (IV-A, IV-B.1, IV-B.2) with respect to the postpartum period, HPV status and cytology (normal vs lesion). However, no significant differences were observed in any of the comparisons (Supplementary Tables 1 and 2).

### Analysis of lesion biomarkers

**Figure 3:**
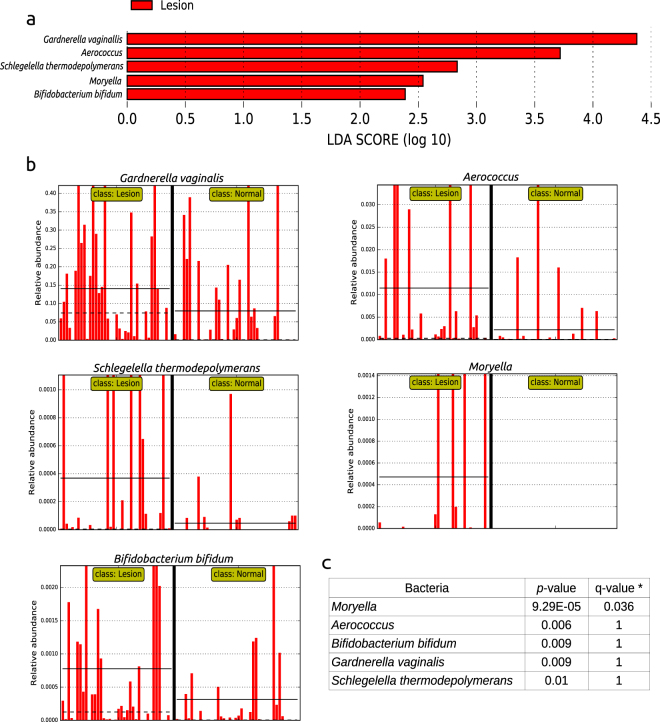
Analysis of cervical lesion putative biomarkers using LEfSe. (**a**) Histogram of the LDA scores (log 10) showing bacteria that presented higher relative abundance in cervical lesions (red) when compared to normal cytology. Only statistically significant differences are shown. (**b**) Histograms showing the relative abundance of the five distinct taxa for each sample in the lesion and normal groups, separated by a thick black line. The solid and dotted black lines in the graphs indicate the mean and median relative abundance values for each group, respectively. The relative abundance (y-axis) is represented as a fraction of 1. (**c**) The p-values obtained by the LfESe analysis and the corrected p-values (q-values) are shown.

We carried out analyses to identify differences in the microbiota composition, independent of the CST classification, through the linear discriminant analysis (LDA) effect size (LEfSe) algorithm as a way to discover potential biomarkers linked to a lesion status. The LEfSe analysis comparing the microbiota between normal and lesion samples showed significant differences in the abundance of *Gardnerella vaginallis*, *Aerococcus*, *Schlegelella thermodepolymerans*, *Moryella* and *Bifidobacterium bifidium*, all present at higher abundance in lesions (Fig. 3). When we performed multiple testing correction to the LEfSe analysis (Fig. 3c), only *Moryella* remained significant (q-value = 0.036). However, due to the fact that the LEfSe algorithm still suggests significant associations (albeit uncorrected for multiple comparisons), we decided to keep all five bacterial taxa in further analysis for confirming their association with cervical lesions.

We calculated odds ratios (OR) to estimate the risk of carrying the bacterial taxa found in the previous analysis in cytological lesions. *Schlegelella thermodepolymerans* and *Moryella* presented ORs of 3.5 (1.4–8.9) and 40.1 (2.28–706), respectively, in lesions compared to normal samples (Table 3). These associations were confirmed as significant with the Chi-square test (Table 3). Overall, our results suggested that specific bacteria, albeit at low abundance, may to be significantly associated with cytological lesions.

**Table 3:** Odds ratio (OR) for the occurrence of specific bacteria in cervical lesions.

Bacteria	Normal % (N/Total)	Lesion % (N/Total)	p-value^1^	q-value^2^	OR (CI95%)^3^	p-value^4^	Adjusted OR (CI95%)^5^	Adjusted p-value^5^
*Gardnerella vaginalis*								
Presence	46 (37/80)	48 (38/80)	0.028	0.14	11.3 (0.6–211.46)	0.10	NA	NA
Absence	6 (5/80)	0 (0/80)						
*Bifidobacterium bifidum*								
Presence	35 (28/80)	39 (31/80)	0.13	0.65	2.2 (0.8–6.3)	0.13	2.88 (0.9–9.1)	0.07
Absence	18 (14/80)	9 (7/80)						
*Moryella*								
Presence	0 (0/80)	15 (12/80)	<0.01	<0.01	40.1 (2.3–705.8)	0.01	NA	NA
Absence	53 (42/80)	33 (26/80)						
*Schlegelella thermodepolymerans*								
Presence	14 (11/80)	26 (21/80)	0.01	0.04	3.5 (1.4–8.9)	0.01	3.44 (1.3–8.9)	0.01
Absence	39 (31/80)	21 (17/80)						
*Aerococcus*								
Presence	50 (40/80)	48 (38/80)	0.17	0.85	4.8 (0.2–102.2)	0.32	NA	NA
Absence	3 (2/80)	0 (0/80)						

^1^Pearson’s chi-square test. ^2^Pearson’s chi-square test after Bonferroni’s correction. ^3^Odds ratios were calculated with Haldane’s modification, which adds 0.5 to all cells to accommodate possible zero counts. ^4^Calculated according to Sheskin, 2004. ^5^Calculated by logistic regression. The OR were corrected by age, smoking, contraceptive methods and number of sexual partners. NA (not available) depicts variables for which calculation of logistic regression is not possible due to the existence of zero counts in one or more categories.

**Figure 4:**
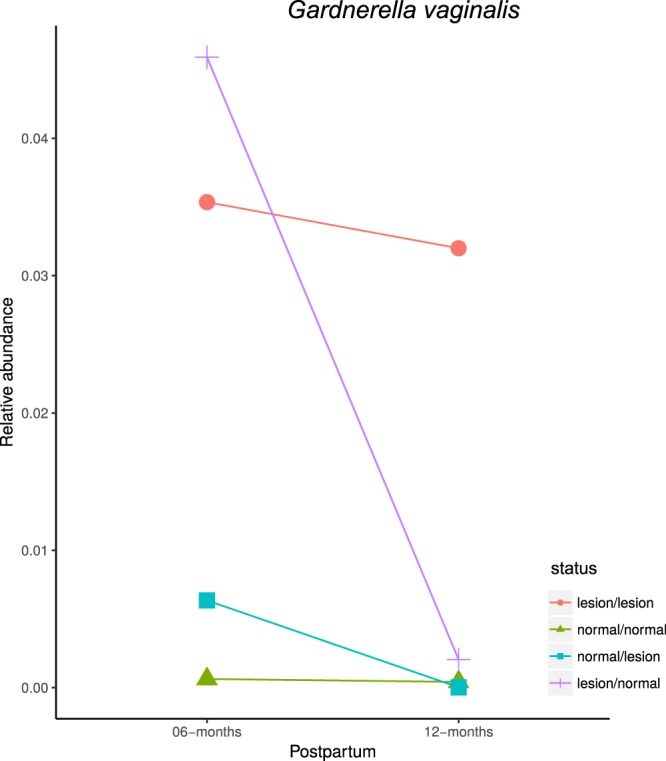
*Gardnerella vaginalis* abundance longitudinal analysis at six and twelve months postpartum in paired samples. The median relative abundance at six and twelve months postpartum of the paired samples that regressed (purple), progressed (blue), or maintained normal (green) or lesion (pink) cytology results is shown. The Wilcoxon test was performed with 95% confidence interval, and the p-value was only significant in the regression group (p = 0.043).

We further analyzed the bacterial taxa that originally displayed significance in the LEfSe analysis in paired samples of the same individuals (6 and 12 months postpartum) for their association with the lesion status. A total of 25 women had longitudinal samples available to analyze; 16 maintained status (normal or lesion at both time points), 5 had lesions that regressed (lesion at the first time point and normal at the second), and 4 had lesions that progressed (normal at the first time point to lesion at the second). Only *Gardnerella vaginalis* presented significant differences in its relative abundance in samples that regressed the cytological status, showing a higher abundance in lesions compared to normal cervices of the same subjects (Fig. 4, Supplementary Figure 3).

## Discussion

HIV-infected women present a higher risk of HPV infection, persistence and development of cervical cancer compared to HIV-negative counterparts^21^. In the present study, we showed that HIV-positive women with HPV infection at 12 months postpartum presented a higher frequency of cervical premalignant lesions than at 6 months postpartum. The persistence of HPV infection is an essential but not sufficient factor for the development of CIN and cervical cancer, and recently the cervical microbiota dysbiosis has been proposed as a key player in the development of cervical diseases^2,10^. Several studies showed an association of high-diversity cervical microbiota with HPV infection, CIN, cervical cancer and HIV acquisition^14,22,23^. However, the profile of such microbiota in HIV-infected women in the postpartum period remained largely unknown.

In this study, we identified four CSTs in postpartum HIV-positive women, which for the sake of definition in previous reports have been named CSTs III, IV-A, IV-B.1 and IV-B.2. CST III is dominated by *L*. *iners*, while CSTs IV (A and B) are high-diversity communities dominated by anaerobic bacteria. We did not find CSTs I, II and V described in previous studies which are dominated by *L*. *crispatus*, *L*. *gasseri* and *L*. *jensenii*, respectively^3^. *L*. *crispatus* (CST I) is present in high frequency in the vaginal microbiota of healthy white (European) and Asian women^3,24^. The absence of this CST among our samples is congruent with the observation that *L*. *crispatus* is associated with reduction in HIV acquisition and is generally absent in HIV-infected women^22^. Furthermore, the composition of CSTs is likely different in distinct ethnic groups. Studies showed that women of African ancestry have a higher prevalence of CST IV when compared to those of Asian and European ancestries^20,24^. The Brazilian population is highly mixed due to its colonization history, with large admixing of European and African descendants^25,26^. Therefore, the ethnic background of the participants in this study may also explain the high proportion of CST IV (A and B) found in the samples.

CSTs IV-A and B are characterized by a high diversity of bacteria and modest to absent proportion of *Lactobacillus sp*., while CST IV-B shows a specific high proportion of *Prevotella*, *Gardnerella*, *Atopobium*, *Mobiluncus* and other bacterial taxa^11,19,20^. Herein, we describe a further stratification of CST IV-B into IV-B.1 (*Gardnerella*-dominant) and IV-B.2 (*Prevotella*-dominant). These two CSTs formed two distinct clusters, but no significant differences were observed in their diversity by Shannon index and PD whole tree analysis. Additionally, CST IV has been associated with HPV infection, CIN and HIV acquisition^14,22,23^. In this study, we showed that CST IV comprised a high proportion (59%) of the samples, but distinct CSTs were not associated with HPV status, the presence of CIN or the postpartum period (6 *versus* 12 months). A previous study reported a high frequency of CST IV among HIV- and HPV-positive women^27^. Additionally, HIV-positive women do not show significant variation in their microbiota irrespective of their HPV status^27^. Our data are in agreement with previous reports^12,22,27^ and suggest that HIV-positive women present a stable community of high-diversity bacteria in the postpartum period that is not altered by CIN status, HPV infection or time postpartum. Addionally, our results are consistent with a study that analyzed the microbiota from an HIV-negative women cohort with normal cytology, which showed a persistent and highly diverse microbiota in the postpartum period of samples collected for up to one year after delivery^28^. On the other hand, another study of vaginal microbiome among European HIV-negative women showed an increased prevalence of CST IV with increasing severity of cervicovaginal lesions, irrespective of HPV status^29^. Our findings prompt further investigation on the stability of microbiome in HIV-negative women with cervical lesions at different time points postpartum compared to HIV-positive women.

Since the CSTs found herein were stable over time, we performed an association analysis of individual bacteria with cervical lesions. We found four bacterial genera in low abundance (*Bifidobacterium*, *Moryella*, *Schlegella and Aerococcus*) and one in high abundance (*Gardnerella*) associated with cervical lesions in initial analyses. The *Moryella* and *Aerococcus* genera belong to the Firmicutes phylum. Recent studies associated this phylum with colorectal cancer^30,31^, but the role of these bacteria in cervical cancer remains unknown. The *Aerococcus* genus modulates the immune response by the production of proinflammatory cytokines^12^. *Moryella* was first identified in abscesses of clinical samples^32^, suggesting it may also play a role in immune modulation. Additionally, *Gardnerella vaginalis* is associated with high-diversity microbiome and is also able to modulate the production of proinflammatory cytokines in the cervical region^12,22^. Whereas inflammatory responses are required to eliminate sexually transmitted infections effectively, a hyperactivated chronic inflammation in the cervix has been correlated with increased risk of CIN and HIV acquisition^12,22,33^.

*Bifidobacterium* has been previously reported in vaginal samples of healthy women^34^. Recent studies described this genus with an antitumor activity, preventing tumor grown and present only in low frequency in colorectal cancer^35,36^. We found herein a positive association of this bacterium with cervical lesions, but its role in the cervical microbiota remains to be elucidated. The *Schlegella* genus was additionally shown in a positive association with lesions, however their role in the cervix is also unknown, and no additional reports are available for comparison. Studies showed the presence of bacterial DNA during sample preparation with DNA extraction kits and other laboratory reagents^37–39^. The presence of *Schlegella* has been previously reported in negative controls of experiments, and it has been suggested that this bacterium is present as contaminant DNA in nucleic acid extraction kit reagents^40^. However, all samples this study were treated with the same reagents, and the presence of *Schlegella* was only observed in samples with cervical lesions. Further studies are therefore necessary to evaluate the association of this bacterium with premalignant cervical lesions.

The five genera originally associated with cervical lesions in the analysis depicted above had their OR estimated for their presence in comparison with normal cytology. The OR of the *Moryella* and *Schlegella* genera were highly significant, of 40x (2.28–706) and 3.5x (1.36–8.9) respectively, whereas the three other genera were not significant. These results and the Chi-square statistics reinforce the strength of the association of these two bacteria with cervical lesions.

The availability of longitudinal samples from many study participants (at 6 and 12 months postpartum) allowed us to evaluate the robustness of particular bacteria as biomarkers for cervical lesions. Upon longitudinal analysis of the taxa that were significantly associated with lesions in the former tests, none of them varied significantly among women that remained with the same status (normal or lesion) over the two time points analyzed (Fig. 4). On the other hand, for those women that progressed from normal to lesion (n = 4) or regressed from lesion to normal (n = 5), only *Gardnerella vaginalis* significantly changed its relative abundance in women in which the lesion regressed. However, among women who changed from normal cytology to cervical lesions, *G*. *vaginalis* levels remained statistically unchanged. These data may suggest that *G*.*vaginalis* may either induce the developmnent of cervical lesions or may colonize the cervix after lesions develop, and our data cannot discriminate between these two possibilities. The lack of additional associations may be due, at least in part, to the small number of paired samples analyzed in each category.

The cervical microbiota displays a complex interaction with the local environment and with the immune system, playing a central role in the cervical homeostasis. Here we demonstrated that the cervical microbiota communities of HIV-positive women at postpartum are stable, generally presenting a high diversity of bacteria and a lack of *L*. *crispatus* dominant types. Moreover, we found three bacterial genera associated with cervical lesions (*Moryella*, *Schlegella and Gardnerella*) through distinct analyses. Little is known about the function of these bacteria on cervical microbiota homeostasis, and it is not clear how these bacteria modulate the development of cervical cancer, whether they arise before or after cervical lesions appear and whether they are a necessary component for the development of malignancy. We propose that distinct microbiota species may serve as sensors for changes in the cervical microenvironment, being able to modulating it or being modified by it towards health or disease.

## Methods

### Sample collection

Eighty women enrolled at the Program for HIV-infected Pregnant Women at the Federal University of Rio Janeiro (UFRJ), Brazil, participated in this study. HIV-positive serological status was confirmed by an HIV rapid or ELISA test and subsequent Western blot following recommendations for HIV diagnosis by the Brazilian Ministry of Health. All participants were under antiretroviral treatment at the time of sample collection. Cervical smear collection was carried out from 2009 to 2010, and two time points were collected for each patient, at six and 12 months postpartum, respectively. However, both time points have been retrieved only for 25 subjects, totaling 50 (25 × 2) paired samples, that were used in the longitudinal analyses. As selection criteria for the longitudinal analyses, we selected samples of subjects with cytology information in the two time points collected. Individuals with ASCUS cytology at any one of the time points were excluded from the analyses. For 80 subjects, unpaired (single timepoint) samples were used in the analysis; the 6-month postpartum time point was available for 26 women, and 12-month timepoint was available for 54 women. Overall, a total of 105 individual samples have been analyzed.

Cytological Pap smear results were obtained from patients’ medical charts and were classified according to the 2001 Bethesda reporting guidelines^41^. Clinical, obstetrical and sociodemographic variables were also retrieved from the medical records and from a questionnaire answered by the participants. All participants of this study were mixed-race people of European and African ancestries. The study was approved by the Ethical Committees of UFRJ (protocols #029/08 to Clementino Fraga Filho University Hospital and #18/10 to Martagão Gesteira Childcare and Pediatrics Institute) and of the Brazilian National Cancer Institute (INCA) (protocol #142/10). All participants signed an informed consent before enrollment in the study. All experimental procedures involving sample collection were performed in accordance with the Brazilian National Ethics Committee guidelines and regulations.

### DNA extraction and HPV typing

Sample collection was performed with two endocervical cytobrushes; one was placed in 100% ethanol for Pap smear diagnosis, while the other was placed into 1 ml of phosphate-buffered saline and stored at −80 °C for DNA extraction. Total genomic DNA was extracted from cervical samples with the RBC Genomic DNA Extraction Kit (Realbiotech, Taiwan), according to the manufacture’s protocol, and was stored at −20 °C until use. HPV DNA was detected by PCR amplification of an L1 gene fragment using consensus primers MY09/MY11 and GP5/GP6, and reaction conditions previously described^42^. All PCR products were analyzed by electrophoresis on 1.5% agarose gels, and positive samples were purified with the Illustra GFX PCR DNA and Gel Band Purification Kit (GE Healthcare Life Sciences, Chicago, IL) and sequenced by Sanger. The sequences obtained were analyzed through BLAST search, and HPV types were assigned based on the HPV type that best matched the query samples.

### Bacterial 16S PCR and analysis

The V3-V6 region of the bacterial 16S rRNA gene was PCR-amplified using the primers 338F and 1061R, which have been reported to amplify over 90% of the bacterial sequences present in the Greengenes database^43^. PCR reactions were carried out using 5 *μ*l Taq 10X buffer, 1.5 *μ*l 50 mM MgCl_2_, 0.4 *μ*l 25 mM dNTP mix, 25 pmol/*μ*l each primer and 0.3 *μ*l Taq Platinum polymerase (5U/*μ*l). The final volume was adjusted to 50 *μ*l with RNase/DNase free water (Life Technologies, Carlsbad, CA). An initial denaturation step of 95 °C for 5 min was carried out, followed by 35 cycles of denaturation (95 °C, 30 s), annealing (59 °C, 30 s) and extension (72 °C, 1 min), and a final elongation step of 6 min at 72 °C. PCR products were visualized by electrophoresis in 1.5% agarose gels and bands of the expected size of 700–1,000 bp were purified with the Illustra GFX PCR DNA and Gel Band Purification Kit. Purified products were quantified in a NanoDrop apparatus (Thermo Fisher Scientific, Waltham, MA), and 0.4 ng of DNA was used to prepare each library with the Nextera XT DNA Sample Preparation kit (Illumina Inc., San Diego, CA). Library concentrations were assessed by qPCR with the KAPA Library Quantification kit (KAPA Biosystems, Wilmington, MA). Libraries were diluted to a final concentration of 10 pM, loaded onto the flow cell and clonal clustering with cBot (Illumina Inc.) was carried out. Sequencing by synthesis was performed on an Illumina HiSeq. 2500 system (Illumina Inc.).

The BCL2FastQ2 Conversion Software (version 2.18, Illumina Inc.) was used to demultiplex data and convert BCL files to FASTQ file formats. Formatted reads were subjected to the FastQC (Babraham Bioinformatics, Cambridge, CBE) for quality filtering using the split_libraries_fastq.py script of QIIME^44^. Operational taxonomic units (OTUs) were assigned to the reads using QIIME’s pick_closed_reference_otus.py script against the Greengenes Database, and the Uclust algorithm was used to group and assign taxa^45^. Sequences were clustered into OTUs using a 97% similarity threshold. OTUs with a number of sequences lower than 0.005% of the total number of sequences generated were discarded and those remaining were summarized at the species level. A rarefaction curve was constructed for each sample (Supplementary Figure 4). The reconstruction of 16S consensus sequence was performed for a partial set of samples using EMIRGE^46,47^ and taxa were assigned to consensus sequences using Blastn against the Greengenes Database. The resultant Blastn top hit (according to sorting criteria of the lowest e-value, highest bit-score, highest percent identity and longest alignment length) was used for classification. The taxa presented and their relative abundances as estimated in EMIRGE were reported and compared to the results obtained in QIIME for a subset of the samples to investigate possible divergence in taxonomic assignment between the two methods.

Unsupervised hierarchical clustering based on Bray-Curtis dissimilarity and average linkage was applied to define clusters according to abundance and taxa diversity of each sample. The clusters found were associated with vaginal microbiome CSTs classified in previous studies^11,19,20^. The alpha diversity of each defined CST was estimated using the Shannon index and the Phylogenetic Diversity (PD) whole tree methods. Clusterization, diversity analyses and plots were carried out using the R environment.

### Statistical analyses

Patients’ sociodemographic and clinical data have been summarized with frequency and medians. Frequencies of cervical cytology results and HPV status were compared between the two timepoints studied (6 and 12 months postpartum) using the Pearson’s chi-square test in the SPSS environment (IBM Corporation, Chicago, IL). The same test was used to compare the distribution of CSTs between different cervical cytology results (normal and LSIL/HSIL), between different HPV statuses (HPV+ and HPV−) and between the two time points postpartum (6 and 12 months). Shannon indexes of the different CSTs were compared using the Mann-Whitney test in SPSS. The Linear Discriminative Analysis (LDA) Effect Size (LEfSe) program of the Galaxy environment^48^, which uses Kruskal-Wallis and estimates the effect size of the comparisons, was used for evidencing potential biomarkers that distinguish normal from lesion samples. A 99% confidence interval has been used in this analysis. We corrected the p-value obtained with LEfEe for multiple testing using the Bonferroni’s correction in the R package. The odds ratio (OR) and 95% confidence interval values for the presence of specific taxa associated with risk of lesions were estimated using Microsoft Excel for Windows, and p-values were calculated according to Sheskin^49^. We calculated the unadjusted OR using the Haldane’s modification, which adds 0.5 to all cells containing zero counts to allow statistical calculations. The OR was adjusted to confounder variables (smoking, sexual partners, ages and contraceptive methods) using logistic regression. The relative abundance of specific bacterial taxa was compared in groups of paired samples that maintained the lesion status (normal or lesion in both samples) or changed the status between samples (normal at 6 months and lesion at 12 months and vice-versa) with the Wilcoxon test in SPSS.

### Data availability

All raw data files are available on Sequence Read Archive (SRA) upon accession number PRJNA392046.

## Electronic supplementary material

Supplementary information
