# Supplementary material for: Analysis of the cervical microbiome and potential biomarkers from postpartum HIV-positive women displaying cervical intraepithelial lesions
Source: Sci Rep. 2017 Dec 12;7:17364. doi: 10.1038/s41598-017-17351-9 (PMC5727204; doi:10.1038/s41598-017-17351-9)
Supplement: Supplementary file 1 — Supplementary information [file 41598_2017_17351_MOESM1_ESM.pdf]

Analysis of the cervical microbiome and potential biomarkers from postpartum HIV-positive women displaying cervical intraepithelial lesions.

Gislaine Curty, Raquel L. Costa, Juliana D. Siqueira, Angela I. Meyrelles, Elizabeth S. Machado, Esmeralda A. Soares, and Marcelo A. Soares.

Supplementary information

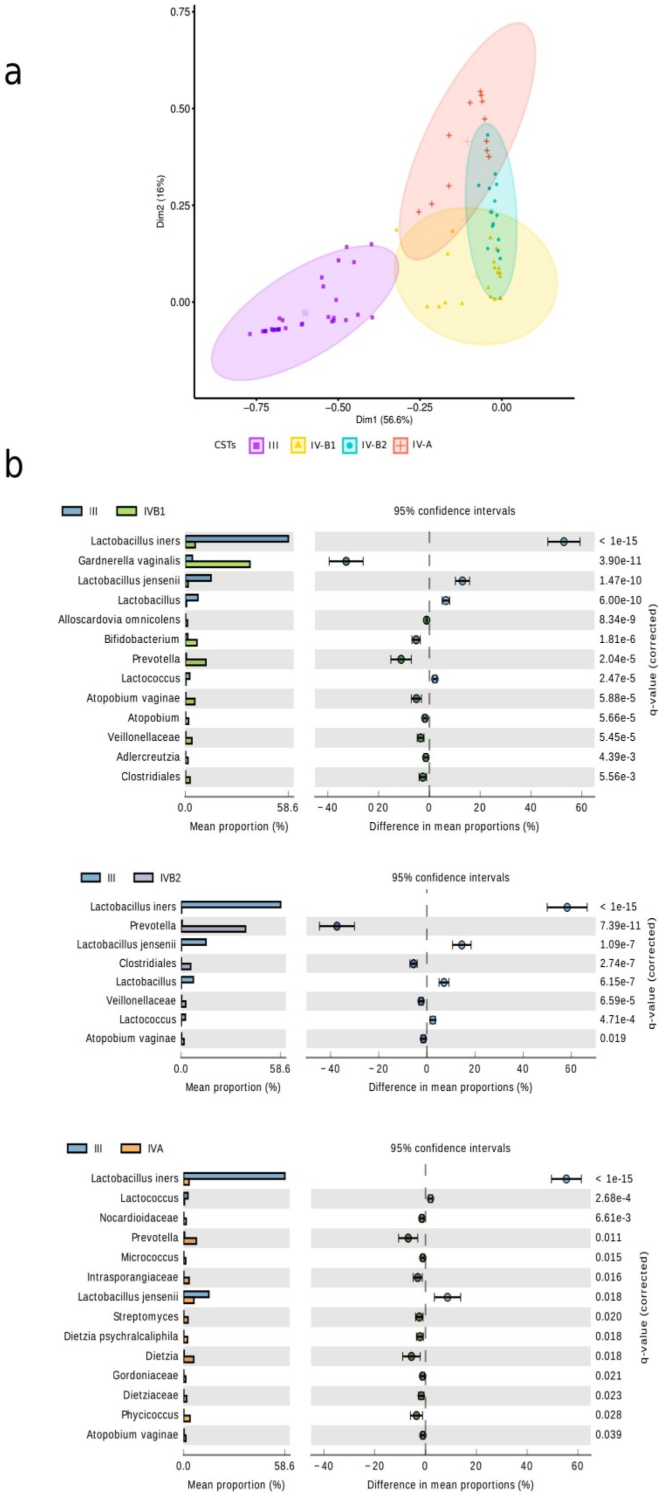

Supplementary Figure 1. Community state type (CST). a) Principal Component Analysis of multi-dimensional data using partitioning methods (k-means) showed four CSTs (III, IV-A, IV-B.1 and IV-B.2). (b) Extended error bar plot showing the differences in mean proportions and CI 95% of bacteria between CST III and each CST IV. The analysis was performed in STAMP using a Student's t test with Benjamini Hochberg correction. Only bacteria with q-values < 0.05 are shown.

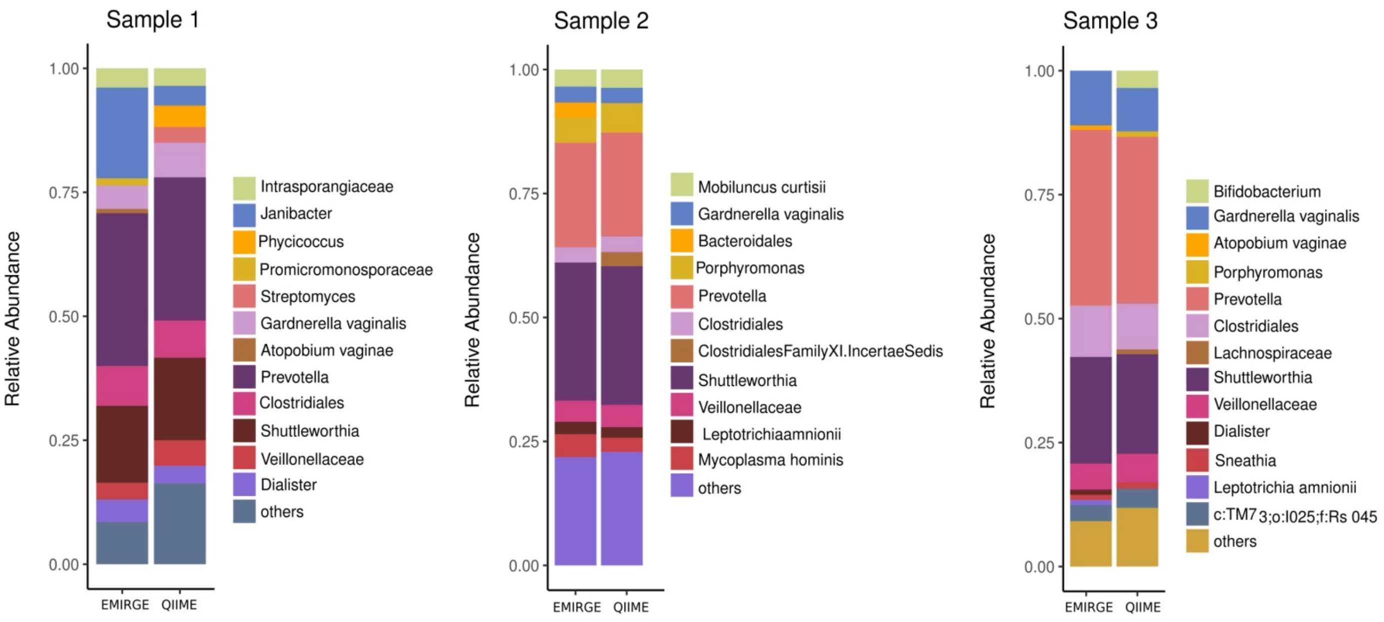

Supplementary Figure 2. Stacked barplot of bacterial taxa relative abundance comparing QIIME and EMIRGE algorithms. The graphs show the relative abundance of based on the 16S gene analysis vs. the QIIME analysis. The Emirge 16S algorithm uses consensus 16S sequences while QIIME uses individual reads. The three samples were chosen at random and the taxa of each sample are shown in the key to the right of bar graph. The Jaccard index was calculated to the samples (1, 2 and 3) and showed similarity of the 61.11 %, 86.36% and 73.68%, respectively, between the methods of analysis.

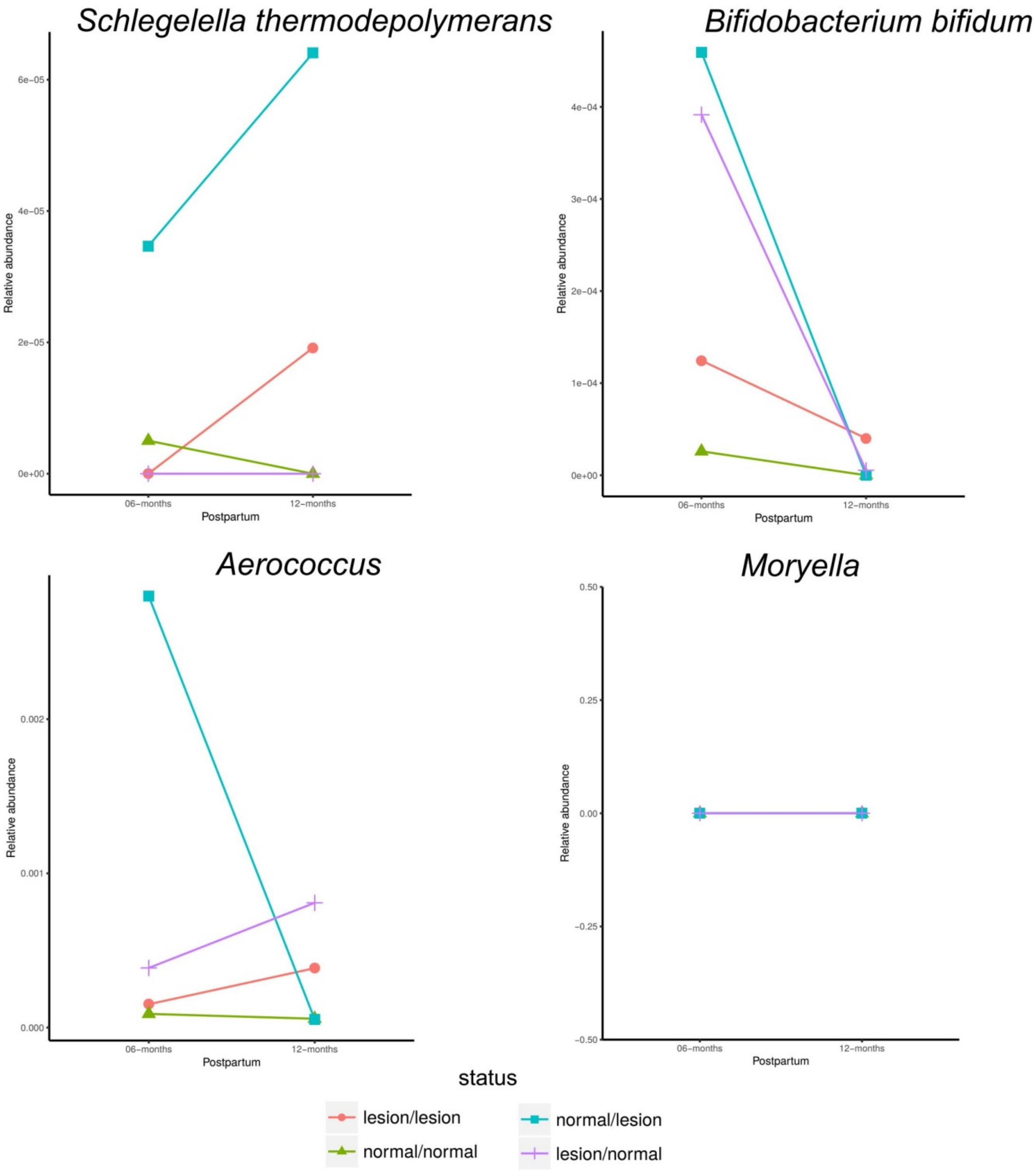

Supplementary Figure 3. Bacterial relative abundance longitudinal analysis at six and twelve months postpartum in paired samples. The graphs show the median relative abundance values of paired samples for four specific bacteria *Schlegelella, Bifidobacterium, Aerococcus and Moryella* in the two collected timepoints (six and twelve months postpartum). The information on the median of samples that regressed (lesion to normal), progressed (normal to lesion) and maintained (lesion or normal) the cytology is shown in the colors and symbols in the graph according to the inset legend at the bottom of the figure.

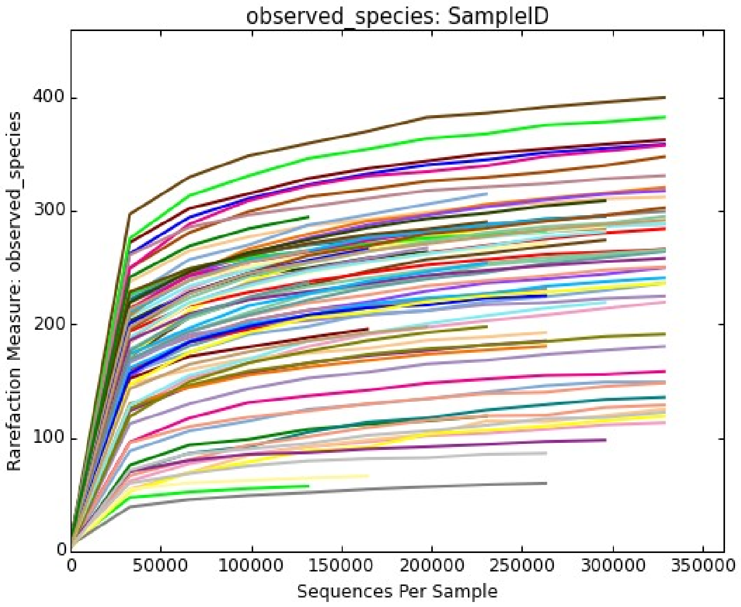

Supplementary Figure 4. Rarefaction curves of a representative set of analyzed samples (n = 80). The graph shows that all samples (represented by individual colored lines) reached a plateau of observed species, suggesting saturated sampling.

Supplementary Table 1. CSTs (III and IV) distribution according to postpartum period, cervical cytology and HPV status.

		CSTIII %(N/Total)	CST IV (N/Total)	p-vaule*
Postpartum	6 months	27 (9/33)	36 (17/47)	0.403
	12 months	73 (24/33)	64 (30/47)	
Cervical Cytology	Normal	54 (24/33)	51 (24/47)	0.759
	Lesion	46 (15/33)	49 (23/47)	
HPV status	Negative	33 (11/33)	23 (11/47)	0.328
	Positive	67 (22/33)	77 (36/47)	

*Pearson's chi-square test.

Supplementary Table 2. CSTs (III, IV, IV-A, IV-B1, IV-B2) distribution according to postpartum period, cervical cytology and HPV status.

		CST III (41%) % (N/Total)	CST IV-A (25%) % (N/Total)	CST IV-B1 (23%) % (N/Total)	CST IV-B2 (11%) % (N/Total)	p-value*
Postpartum	6 months	11 (9/80)	6 (5/80)	10 (8/80)	5 (4/80)	0.444
	12 months	30 (24/80)	19 (15/80)	13 (10/80)	6 (5/80)	
Cervical Cytology	Normal	23 (18/80)	16 (13/80)	10 (8/80)	4 (3/80)	0.376
	Lesion	19 (15/80)	9 (7/80)	13 (10/80)	8 (6/80)	
HPV status	Negative	14 (11/80)	9 (7/80)	3 (2/80)	3 (2//80)	0.298
	Positive	28 (22/80)	16 (13/80)	20 (16/80)	9 (7/80)	

*Pearson's chi-square test.
